# Electrospray ion beam deposition plus low-energy electron holography as a tool for imaging individual biomolecules

**DOI:** 10.1042/EBC20220165

**Published:** 2023-03-29

**Authors:** Hannah Ochner, Stephan Rauschenbach, Luigi Malavolti

**Affiliations:** 1 Max Planck Institute for Solid State Research, Heisenbergstr. 1, 70569 Stuttgart, Germany; 2 Department of Chemistry, University of Oxford, 12 Mansfield Road, Oxford OX1 3TA, U.K.

**Keywords:** low-energy electron holography, native electrospray ion beam deposition, protein imaging

## Abstract

Inline low-energy electron holography (LEEH) in conjunction with sample preparation by electrospray ion beam deposition (ES-IBD) has recently emerged as a promising method for the sub-nanometre-scale single-molecule imaging of biomolecules. The single-molecule nature of the LEEH measurement allows for the mapping of the molecules’ conformational space and thus for the imaging of structurally variable biomolecules, thereby providing valuable complementary information to well-established biomolecular structure determination methods. Here, after briefly tracing the development of inline LEEH in bioimaging, we present the state-of-the-art of native ES-IBD + LEEH as a method of single-protein imaging, discuss its applications, specifically regarding the imaging of structurally flexible protein systems and the amplitude and phase information encoded in a low-energy electron hologram, and provide an outlook regarding the considerable possibilities for the future advancement of the approach.

## Introduction

The imaging of biomolecules is of great importance for understanding biological systems as a biomolecule’s structural features are intricately related to its function [1–5]. At present, the leading methods employed for structure determination of biomolecules are X-ray crystallography, nuclear magnetic resonance spectroscopy (NMR), and electron cryo-microscopy (cryo-EM) [6–10], which provide high-resolution molecular models by averaging over data acquired from a large number of molecules in purified and enriched samples. The near exponential growth of available protein structures in the Protein Data Bank (PDB) bears witness to the success of these techniques. The need for averaging, however, imposes limitations on the types of molecules that can be imaged as well as on the sample preparation process [11,12]. In particular, averaging impedes the imaging of molecules with a high degree of conformational variability, since structural diversity on the level of individual molecules cannot be adequately represented in an image derived from an ensemble. To overcome these limitations and further advance the reach of biomolecular imaging, different approaches are currently being explored. One potentially universal solution would be the high-resolution imaging of individual molecules, revealing unaveraged molecular structures and thereby mapping the conformation space of the molecule. Such true single-molecule imaging methods include scanning probe microscopy (SPM), and coherent diffraction imaging using an X-ray free electron laser (XFEL) [13–18]. An emerging, potentially high-resolution imaging method for individual molecules is low-energy electron holography (LEEH) [19–32]. Even before the breakthrough experiments in molecular structure determination were carried out in the early 1950s for both DNA and proteins [33–36], Dennis Gabor proposed an electron microscopy approach in which the superposition of a wave scattered from an object and an unscattered reference wave forms an interference pattern (the hologram), which encodes the object’s structural information [37]. His ‘new microscopic principle’ [37], which became known as holography, would be implemented in the form of a lens-less microscope and thus overcome the resolution limit imposed by the aberrations in the electron lenses that were hampering the widespread application of electron microscopes at the time. Additionally, the method would give access to the relative phase information between scattered wave and reference wave, which would allow for the reconstruction of an image from a single object instead of a periodic crystal [37–39], thus making it a promising candidate for single-molecule imaging.

Holographic imaging can be realised with any kind of coherent radiation in different imaging geometries: inline [19–32,37,38,40] and off-axis [40–44], depending on the availability of suitable optical elements for the type of radiation employed. However, biomolecular imaging on the single-molecule scale requires high contrast. To achieve this, high scattering cross-sections are necessary, which occur at low electron energies in the range of 100 eV [45,46]. While there have been demonstrations of the imaging of biological samples using high-energy electron holography implementations in transmission electron microscopy (TEM) [47,48], the imaged structures are in general on a larger spatial scale. As the present paper focuses on holographic imaging of individual biomolecules on the nanometre and sub-nanometre level, a detailed discussion of high-energy electron holography implementations is beyond the scope of this article, for an overview of the topic we refer the reader to [43,47,49,50].

The application of low-energy electron holographic imaging to individual biomolecules first became feasible in the wake of the development of highly coherent low-energy electron sources in the form of ultrasharp tips [19,20,51]. While this enabled the experimental acquisition of low-energy electron holograms of biological samples suspended over a holey substrate [25,26,28,52–56], the discovery of graphene [57] significantly expanded the applicability of the method as it provided a continuous sample support that facilitates the use of low-energy electrons in transmission [58,59], can be prepared in an ultraclean way [60], and creates a well-defined reference wave. This not only allows the imaging of smaller objects but also simplifies hologram reconstruction, i.e. the retrieval of real-space images of the objects. Finally, combining LEEH with a highly controlled sample preparation performed by electrospray ion beam deposition (ES-IBD) [61] recently allowed the sub-nanometre-resolution imaging of native proteins at the single-molecule level [31,32].

Here, we review the development and application of inline LEEH for the single-molecule scale imaging of biological molecules. We first present the principles of holography and discuss the implementation of a LEEH microscope in conjunction with ES-IBD as the sample preparation method. Subsequently, we summarise its application as a single-molecule imaging method for biomolecules, demonstrating that LEEH can map the conformational space of flexible proteins, and thus, for samples prepared by ES-IBD, provide information about the molecules’ gas-phase structure. Additionally, we report that the application of phase reconstruction algorithms allows for the retrieval of further structural details that can be traced to local changes in the mean inner potential of the molecules as well as to the presence of local electric fields. Following this, we provide an outlook regarding the future development of the technique.

## LEEH

Although LEEH has only recently been established as a tool for single-molecule imaging of biomolecules [29,31,32], the underlying idea of holographic imaging has been proposed by Gabor in 1948 [37]. Given that such an imaging scheme can be realised without the use of lenses or other optical elements (inline geometry), the obtainable resolution is in principle only diffraction-limited [62,63], i.e. dependent on the wavelength and coherence of the irradiating beam. Since coherent illumination is necessary for creating holograms, the development of the method received a boost with the advent of laser sources, which allowed holography to be widely applied in optical microscopy and related techniques [64–66].

In parallel, holography has also been developed as an electron microscopy technique, mostly in the context of TEM applications in materials science [43,67,68]. Nowadays, aberration-free lenses are available not only in photon optics but also in high-energy electron imaging [69]; hence for these applications, a holographic approach is not needed to achieve Gabor’s original goal of aberration-free imaging. Nevertheless, the holographic method is very well suited for quantitative phase imaging, as demonstrated in a wide variety of implementations ranging from holographic imaging in TEM [42,44,70] to X-ray holography applications [71,72] in inline [71,72], off-axis [42,44,70], and Fourier transform holography [73] geometries, and is thus of interest for many applications, such as the imaging of biological systems or the mapping of electromagnetic fields. This success of holographic phase imaging also indicates that method development for LEEH can profit from focusing on single-molecule level phase reconstruction.

**Figure 1:**
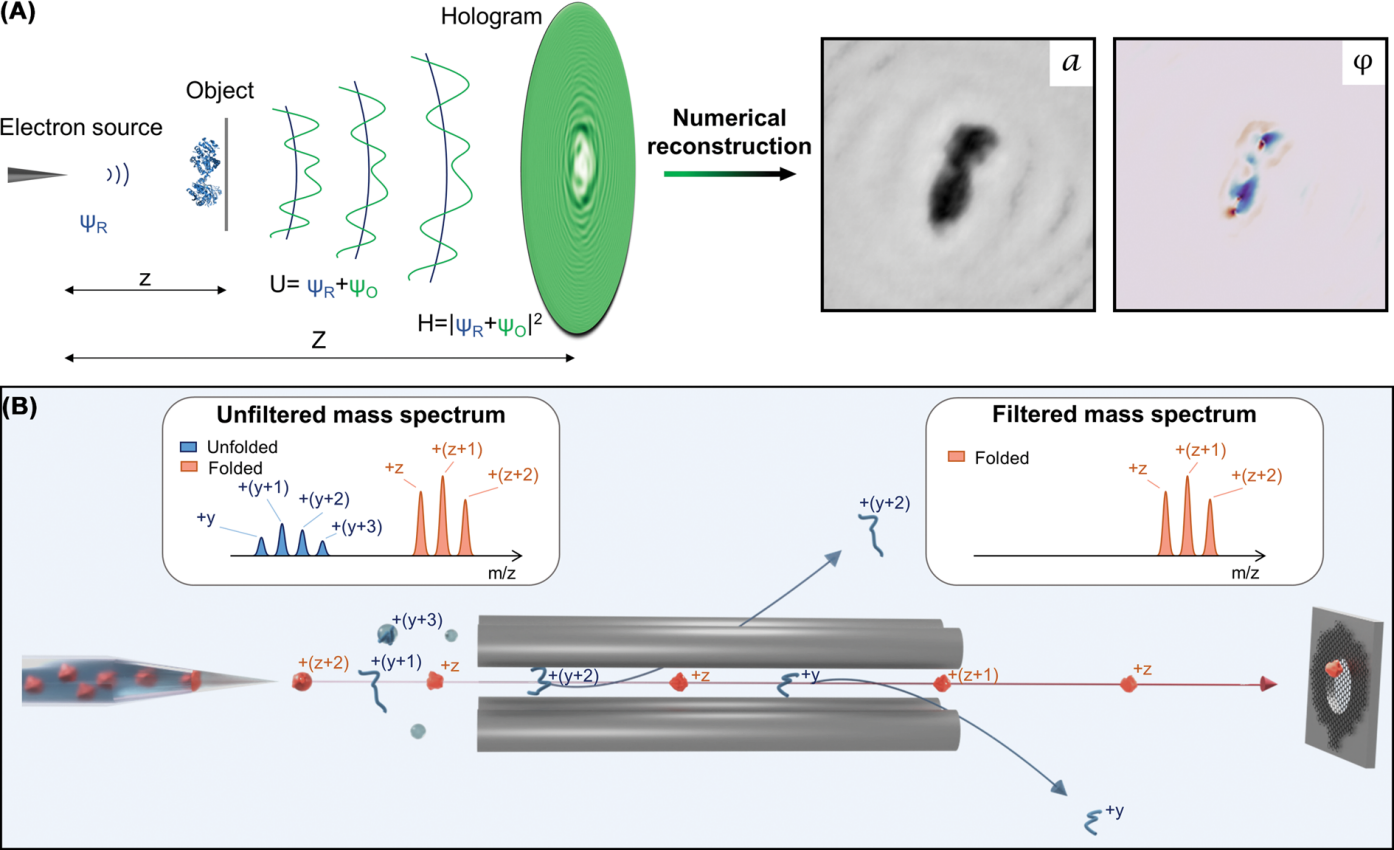
Experimental methods (**A**) Schematic of the inline LEEH experimental setup, consisting of an electron source, proteins deposited on a free-standing single-layer graphene (SLG) substrate (object) and a detector to record the hologram. The hologram (*H*) is created as the interference pattern between the wave scattered by the object (Ψ*_O_*) and the incident reference wave (Ψ*_R_*) and is subsequently numerically reconstructed to obtain real-space amplitude and phase images. The magnification is determined by the ratio of *z* and *Z*. Figure adapted from Ochner et al., used under CC BY-NC-ND [120]. (**B**) Sketch of the native ES-IBD sample preparation process, during which the molecules proceed from solution through the gas phase to soft-landing deposition. Depending on their folding state, the molecules assume different charge states (labelled +y and +z). Before deposition, the relevant species is selected by mass spectrometry.

Since the single-molecule level imaging of biomolecules requires high contrast and thus the use of low-energy electrons, the lack of aberration-free electron lenses for this energy range [74] necessitates the implementation of LEEH in Gabor’s original lens-less inline imaging geometry. An inline LEEH setup (Figure 1A), as originally designed by Fink et al. [19], consists of three main parts: a coherent electron source, the sample to be imaged, and a detector to record the hologram. A divergent beam of coherent electrons in an energy range of 30–200 eV (corresponding to wavelengths of 2.23–0.87 Å, which, depending on the numerical aperture of the setup, yields a theoretical resolution limit (Abbe diffraction limit [62,63]) of approximately 1–3 Å) is produced by field emission from sharp tungsten tips [19,20,75,76], forming the incident reference wave Ψ*_R_* that subsequently interacts with the object. This interaction results in the scattered wave Ψ*_O_*. In the area between sample and detector, the two waves interfere, creating the superposition *U* = Ψ*_R_* + Ψ*_O_*, which is recorded at the detector as the hologram *H* =|Ψ*_R_* + Ψ*_O_*|^2^ [37,38]. The amplitude information stored in the hologram can be retrieved by a numerical reconstruction process based on wave field propagation, in which the exit wave *U*(*x,y*) created by the interaction in the object plane is obtained as the solution of a Fresnel–Kirchhoff integral [62,77] (eqn 1):

(1) $$U(x,y)=-\frac{i}{\lambda }\int_{-\infty }^{\infty }\int_{-\infty }^{\infty }H(X,Y)\Psi_{R}(X,Y)\frac{e^{-\text{i}k\rho }}{\rho }dXdY,$$

with $\rho =\sqrt{(X-x{)}^{2}+(Y-y{)}^{2}+(Z-z{)}^{2}}$. (*x,y,z*) denote the co-ordinates in the object plane, while (*X,Y,Z*) are the co-ordinates in the detector plane. Due to the divergence of the beam, the ratio between *z* and *Z* determines the achievable geometric magnification of the inline holography setup (see Figure 1A).

**Figure 2:**
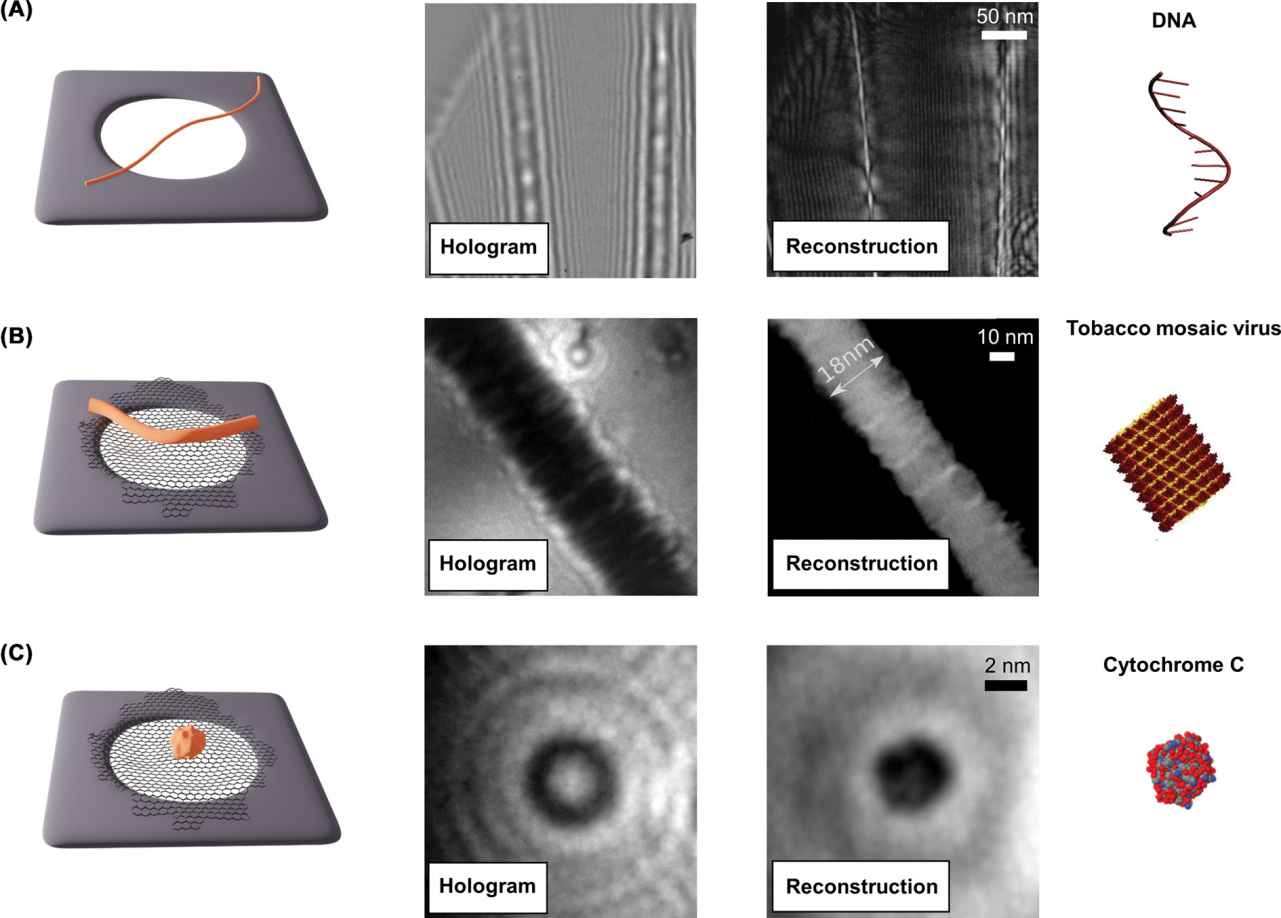
Development of LEEH imaging of biomolecules (**A**) Left to right: Sketch of a free-standing DNA strand stretched over a holey substrate, LEEH hologram, amplitude reconstruction, and model of single-stranded DNA. Hologram and amplitude reconstruction images adapted from Latychevskaia et al., used under CC BY 4.0 [56]. (**B**) Left to right: Schematic of tobacco mosaic virions stretched over a SLG-covered holey grid, LEEH hologram, amplitude reconstruction, and model of a tobacco mosaic virion. Tobacco mosaic virions have been deposited on SLG via a dropcast procedure [29]. The use of SLG as a substrate reduces charging and thus artefacts in the reconstruction. Hologram, amplitude reconstruction, and model adapted from Longchamp et al., with the permission of AIP Publishing [29]. (**C**) Left to right: Sketch of a cytochrome C molecule deposited on a SLG-covered holey grid by ES-IBD, LEEH hologram, amplitude reconstruction, and model of a cytochrome C molecule. The SLG substrate allows the imaging of smaller molecules that do not stretch over a membrane, while sample preparation by ES-IBD provides a clean and controlled method for protein sample preparation in a native-like state on surfaces in UHV. Hologram, amplitude reconstruction, and model adapted from Longchamp et al., used under CC BY-NC-ND [31].

While the use of low-energy electrons has the potential for nondestructive imaging of fragile biomolecules [29,31,78], it also imposes strict requirements on the sample preparation. Because of the small mean free path of low-energy electrons [45,46], substrates and matrices employed in traditional TEM and cryo-EM experiments cannot be used [30]. The development of a reliable procedure for sample preparation, i.e. the identification of a low-energy electron-transparent substrate and controlled molecular deposition methods, has been a critical step in the development of LEEH as an imaging method for biomolecules. Initial LEEH experiments were carried out on free-standing fibres spanned over the holes of a perforated conductive foil [19,22,24–26,28,52–55], i.e. without the presence of a substrate (Figure 2A). Employing this sample-preparation technique, the first LEEH experiments on biological samples, ranging from purple membrane [52] to DNA fibers [25], were reported in the 1990s. Despite this initial success, LEEH imaging was still limited to few specific biological objects and molecular structures that could be stretched over empty holes [25,26,28,54–56,79]. Additionally, the use of free-standing fibres induces biprism distortions that, in LEEH, negatively impact the reconstruction process and the resolution of the reconstructed image [26,80–82].

The introduction of a SLG substrate, which exhibits high transmittance for electrons in the energy range employed in LEEH [30,58,59,83–86], as support for the target objects [58,59] vastly extended the possibilities offered by the LEEH method [29,83] (Figure 2B). The graphene layer provides a uniform equipotential plane, which effectively reduces the undesired biprism effect and artefact-inducing charging effects, thereby creating an undisturbed reference wave. The excellent conductivity of graphene [87] and the fact that electronic states of proteins and graphene show a significant overlap [88] likely are conducive to protecting the molecules from radiation damage caused by ejection of electrons from the proteins, which can swiftly be replaced from the graphene. Additionally, SLG has the advantage of only weakly interacting with biomolecules, which facilitates the preservation of biologically relevant states of the sample.

With graphene being established as a suitable substrate, LEEH single-molecule imaging additionally requires a clean, highly controlled, and ultrahigh vacuum (UHV)-compatible method for depositing fragile biomolecules on the substrate. Native ES-IBD combines all these features and thus is the natural choice of sample-preparation technique for the LEEH imaging of biomolecules. The method is particularly suitable in this context since it can not only transfer complex biomolecules such as proteins onto surfaces in UHV while retaining a native-like state but can, in combination with preparative mass spectrometry, selectively deposit the relevant molecular species (Figure 1B) [89–92] and control the coverage, ensuring a sparse sample that allows for a well-defined reference wave.

**Figure 3:**
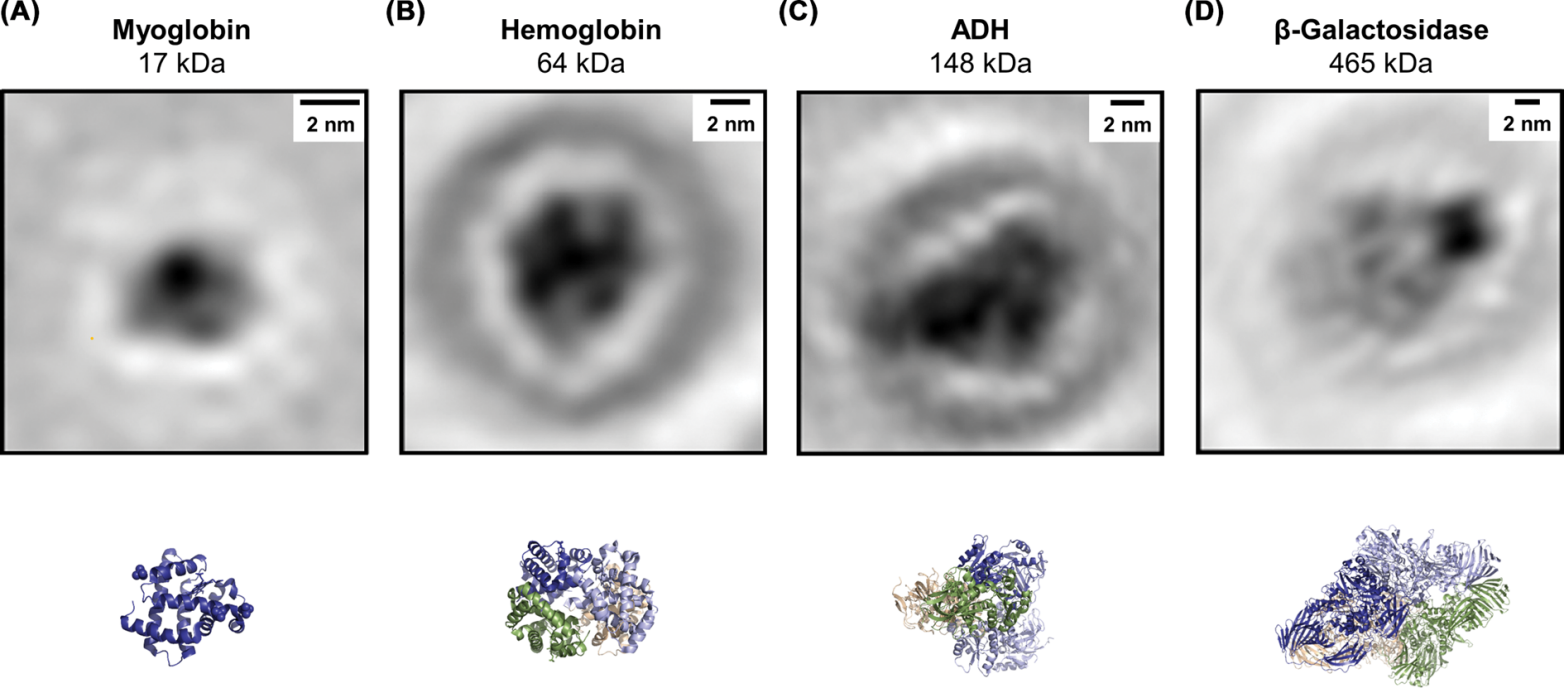
Overview of protein systems imaged by LEEH: amplitude reconstruction of individual molecules with corresponding model (**A**) Myoglobin [93]. (**B**) Hemoglobin [94]. (**C**) Alcohol dehydrogenase (ADH) [95]. (**D**) β-Galactosidase [96]. All proteins were deposited on SLG by native ES-IBD.

The combination of LEEH imaging with ES-IBD-prepared samples forms a potent tool capable of providing access to a diverse range of molecules on the single-molecule scale [31,32] (Figure 2C). To illustrate this, Figure 3 provides an overview of proteins imaged by LEEH after mass-selective deposition by ES-IBD, ranging from small proteins with molecular masses of 10–20 kDa (cytochrome C (12 kDa, Figure 2C [31]), Myoglobin (17 kDa, Figure 3A)) to large proteins with molecular masses of several hundreds of kDa (β-Galactosidase, 465 kDa (Figure 3D)). In all cases, the reconstructed amplitude images match the molecular models in size and shape. In the case of the larger molecules, in particular in the β-galactosidase molecule in Figure 3D, additional substructure is revealed.

In principle, the LEEH imaging of structures both larger and smaller than shown in Figure 3 is possible. In the case of large molecular systems, the limitations will most likely be defined by the ability of low-energy electrons to penetrate the structures as well as by the ratio of object size and the illuminated area, which has to remain in the range of 1% to ensure artefact-free imaging [38,97,98]. The imaging of smaller structures is mainly limited by the current resolution of the technique (approximately 5 Å [32]), which could in principle be enhanced by improving the performance of LEEH imaging, for instance via an increased coherence of the electron emitters [99] or via measurements at cryogenic temperatures [100].

In the light of these results, the combination of ES-IBD and LEEH opens up the possibility of imaging a large range of protein systems at the single-molecule level. Additionally, the method could be extended to the imaging of other classes of biomolecules such as DNA-based structures, or to glycans, which, due to their high degree of conformational variability, pose a particular challenge to averaging-based methods.

## Imaging conformational variability and gas-phase-related conformations

**Figure 4:**
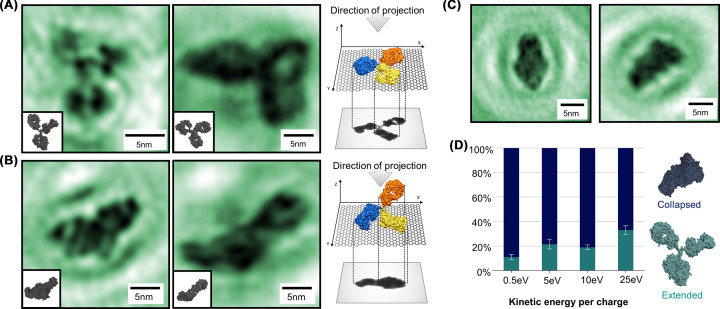
Imaging conformational variability and gas-phase-related conformations (**A**) Amplitude reconstructions of IgG antibody molecules in a flat adsorption geometry, resulting in Y-shaped structures. (**B**) Amplitude reconstructions of IgG antibody molecules in a vertical adsorption geometry, resulting in two-lobe structures. The diversity in the observed structures demonstrates that LEEH imaging can map the conformational space of conformationally variable molecules. (**C**) Examples of gas-phase-related, collapsed antibody structures. (**D**) Percentage of extended and collapsed conformations observed by LEEH imaging depending on the landing energy. At higher landing energies, more extended structures are observed, indicating that the compact structures are related to the collapsed gas-phase conformation from which extended structures can be recovered if sufficient energy is transferred during the landing process. Figure adapted from Ochner et al., used under CC BY-NC-ND [32].

Since single-molecule methods are capable of probing the full conformational space of a molecular system in a given imaging environment, such techniques are of particular interest in the investigation of systems with a high degree of conformational variability [101,102]. The ability of LEEH amplitude imaging to map different conformations has recently been demonstrated in an ES-IBD+LEEH study of IgG antibodies (IgGs) [32], which consist of three subunits connected by a highly flexible hinge region. Figure 4 demonstrates the ability of LEEH imaging to distinguish different antibody conformations, both on the level of the overall molecular shape as well as on the subunit level, and thus to map the conformational space accessible to IgG molecules on an SLG surface. The observed structures can be classified into two main species: extended structures with clearly discernible subunits (Figure 4A,B) and compact structures without discernible substructure (Figure 4C).

The class of extended molecules represented in Figure 4A,B can be further subdivided into molecules with three distinguishable subunits (Y-shaped conformation, Figure 4A) and with two distinguishable subunits (Figure 4B). As shown in the sketches in Figure 4A,B, this distinction can be traced to the adsorption geometry of the molecules with respect to the SLG surface: while the Y-shaped molecules lie flat on the graphene substrate, the two-lobe species can be associated with molecules adsorbed in a vertical geometry. Since LEEH imaging yields 2D projections along the optical axis, the resulting molecular images exhibit shapes with three and two discernible subunits, respectively [32].

The conformational variability of the imaged IgGs is, however, not only visible in the overall molecular shape determined by the adsorption geometry, since the molecules in each subclass, while clearly recognizable as the same conformational species, can differ significantly (Figure 4A,B). This diversity directly traces the molecules’ conformational space since the observed extended conformations can be mapped to the IgG crystallographic structure [103] by a simple rotation of four bonds of the hinge region [32] (insets of Figure 4A,B).

The observed compact structures (Figure 4C), on the other hand, do not match any of the projections obtained from antibodies with an extended hinge region in either of the adsorption geometries discussed in Figure 4A,B. The origin of these compact surface conformations is directly related to the influence of the ES-IBD sample preparation on the protein conformation, which could be altered during several steps within this process. The transitional steps, in which the protein’s environment changes, are particularly likely to induce conformational changes. The final desolvation step when transitioning from the solution phase into the gas phase, which directly affects the hydrophobic and electrostatic interactions within the molecule [104], marks the most drastic change of environment during the ES-IBD process. While mass spectrometry and ion mobility measurements show that IgG antibodies do not unfold or fragment upon transition into the gas phase [105–107], experimentally obtained IgG antibody collision cross-sections are up to 30% smaller than the theoretical values calculated from the crystallographic model [107]. This indicates that flexible proteins such as antibodies can undergo a collapse of the tertiary structure during desolvation [107–111] as also shown by molecular dynamics simulations, which yield collapsed, compact gas-phase structures of IgG antibodies [107] with dimensions matching those of the experimentally observed compact structures. The compact conformations observed by LEEH imaging on ES-IBD-prepared samples are thus interpreted as intact antibodies that have retained their collapsed gas-phase conformation upon deposition on the SLG surface.

Since both ion mobility-based collisional cross-section measurements and molecular dynamics simulations [107] indicate that IgGs assume compact, collapsed gas-phase conformations, the observation of extended IgG structures in LEEH measurements is thus related to the landing process of the molecules on the SLG surface, which is in general associated with an energy transfer from translational kinetic energy into vibrational modes of both the molecule and the graphene substrate [88,112–114]. In order to observe extended antibody conformations on the surface, the energy transfer during the landing process has to allow for the re-extension of the hinge region and thus the recovery of extended conformations from collapsed structures. In general, for landing energies below the reactive regime, an increase in landing energy is correlated with an increase in extended surface conformations [113]. By tuning the landing energy in a range from 0.5 to 25 eV per charge, an increase in the percentage of extended antibody conformations on the surface from 11 ± 2% at 0.5 eV per charge to 33 ± 3.5% at 25 eV per charge (Figure 4D) was observed [32]. This energy dependence further supports the interpretation that the observed compact structures are directly related to the molecules’ gas-phase structure while the extended structures are recovered during the landing process in conditions of favourable orientation and sufficient energy transfer. The possibility to distinguish and, via the tuning of the landing energy, select between extended conformations and compact gas-phase-related conformations allows the study of both conformational spaces by ES-IBD+LEEH. The technique can thus be relevant for exploring questions pertaining to both the native structure of conformationally variable proteins as well as to conformational changes occurring in the gas phase. The imaging of the latter could be of particular interest in the context of structural biology measurements performed in the gas phase.

## LEEH phase imaging

While the exploration of the conformational space of proteins, which relies on the accurate reconstruction of the molecules’ shape and size, can be carried out using a one-step amplitude reconstruction routine [31,32,77], biological matter interacting with low-energy electrons in general does not only induce changes in the amplitude of the incident electron beam but also in its phase [45,46,115]. However, in LEEH, the phase information cannot be accurately reconstructed by a one-step reconstruction process due to the fact that the hologram itself is a real-valued intensity distribution, which only encodes relative phase information, but does not contain the absolute phase values of the complex wave field in the detector plane [116–118]. This results in an ambiguity in the one-step reconstruction by wave field propagation, which leads to the simultaneous reconstruction of the image and its complex conjugate (the twin image) [38,119]. Via out-of-focus contributions, the latter induces inaccuracies in the phase reconstruction, which can be suppressed by employing an iterative phase retrieval scheme as has been shown for simulated inline holograms [119] and been applied in the experimental phase reconstruction of charged impurities on graphene [84].

In high-energy electron imaging, the phase shift induced by the sample can directly be related to its mean inner potential, i.e. the spatially averaged electrostatic potential defined as $V_{\text{mean}}=\frac{1}{\Omega }\int_{\Omega }V(\text{r}){\text{d}}^{3}\text{r}$, where *V*(**r**) is the electric potential of the object in a given volume Ω [43,46]. For an object consisting of atoms of similar electrostatic properties and scattering strength, this implies a relation between the induced phase shift and the object’s thickness or atomic density. Phase imaging has only recently been reported in the context of experimentally acquired inline low-energy electron holograms of biological specimens [120]. The technique hence continues to be developed and some features of the resulting phase maps remain to be further explored on a quantitative level. Similarly to the high-energy case, the LEEH phase imaging results indicate a strong correlation between the experimentally measured induced phase shift and the projected atomic density of individual protein molecules [120]. Given that proteins mainly consist of atoms of very similar scattering strengths, this observed correlation in turn implies that the mean inner potential of the molecules is a major contribution to the phase shift measured by LEEH imaging.

**Figure 5:**
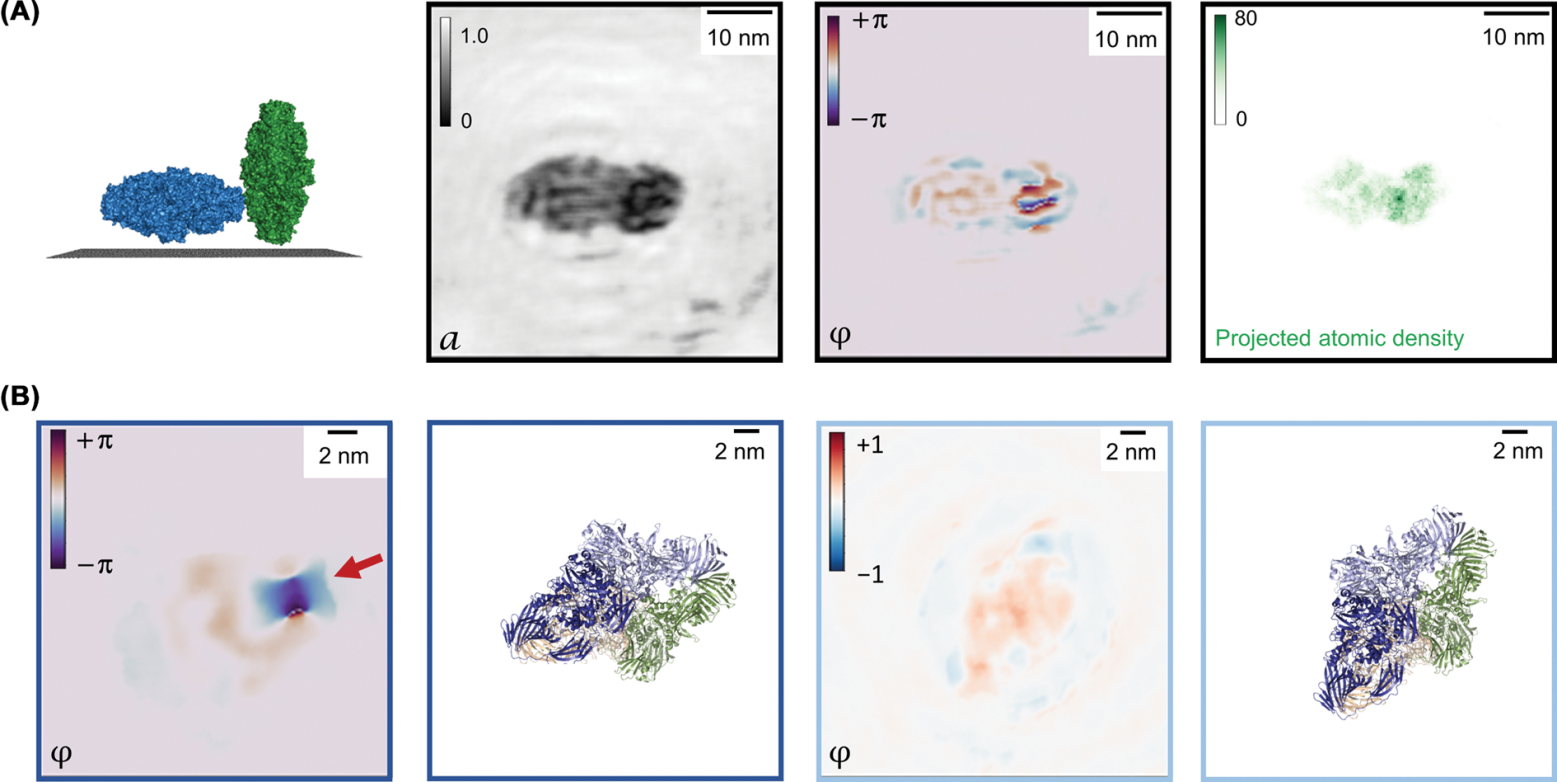
LEEH phase imaging (**A**) Left: Schematic depiction of the orientation of two β-galactosidase molecules, whose amplitude and phase reconstruction is shown in the central panels, with respect to the SLG surface. The different orientations of the molecule result in different molecular thicknesses along the optical axis. Centre: Amplitude and phase reconstruction of a hologram of two β-galactosidase molecules in orientations as depicted on the left. Right: Projected atomic density obtained from PDB models of β-galactosidase (PDB: 6CVM [96]) in the corresponding orientations. The colour scale maps the number of atoms projected into each pixel. The difference in projected atomic density between the two molecules is reflected by the difference in induced phase shift. (**B**) Phase reconstruction of two β-galactosidase molecules in similar orientations with (left, dark blue frame) and without (right, light blue frame) a localised charge in the vicinity of the molecule. The signal from the localised charge dominates the reconstructed phase map. Figure adapted from Ochner et al., used under CC BY-NC-ND [120].

The correlation between induced phase shift and projected atomic density can most clearly be observed when reconstructing holograms of two objects with varying molecular thickness as displayed in Figure 5A, which shows the iterative amplitude and phase reconstructions (centre) along with the corresponding projected atomic density (right) of two β-galactosidase molecules in different orientations with respect to the surface. As shown in the schematic of the molecular orientations in Figure 5A, the molecule on the left (blue) is in a flat orientation with respect to the graphene surface, while the molecule on the right (green) is in an upright orientation, resulting in a difference in molecular thickness along the optical axis and thus in a difference in projected atomic density. The changes in the projected atomic density are reproduced by the measured phase shift, which is much lower in the flat molecule on the left than in the upright molecule on the right. A coarse theoretical estimate of the phase shift induced by a large protein such as β-galactosidase, obtained by summing the phase shifts calculated for individual light atoms (C, N, O) via a partial wave-based calculation [121] according to the amount of atoms in the electron path, yields a quantitative agreement with the measured phase shifts: the calculation yields a phase shift of approximately 0.05 rad per atom [98], corresponding to 0.5–1.5 rad for a molecule in flat orientation (projected atomic density 10–30 atoms per pixel) and 2–4 rad for a molecule in upright orientation (projected atomic density 40–80 atoms per pixel) [120]. This implies that LEEH phase imaging can be used to map projected atomic density and thereby local changes in the mean inner potential of biomolecules. For a full interpretation of the LEEH phase maps, however, further research needs to be carried out since a full description of the interaction of low-energy electrons with biological matter is currently not computationally feasible. Specifically, the simultaneous presence of positive and negative phase shifts, which in some examples persists even after phase unwrapping [120], remains to be explored further.

The sensitivity of LEEH phase to changes in electrostatic potential is further confirmed by the strong phase signatures produced by the electric fields of localised charges on or close to the imaged molecules. Figure 5B shows a comparison of two β-galactosidase molecules in similar orientations. While the molecule on the right, which is not in the proximity of a localised charge, displays a uniform phase shift, the phase reconstruction of the molecule on the left is dominated by a localised feature, which can be associated with a negatively charged feature on the graphene substrate with an estimated charge of 4–5 electron charges [83,120]. This shows that an accumulation of charge strongly affects the LEEH phase reconstruction, which could give an indication of the conditions under which phase shifts of opposite signs may occur in the same image, namely when phase shifts can be attributed to charges of opposite sign or to different origins such as a charge distribution and the mean inner potential of a molecule [120].

In summary, the mapping of changes in mean inner potential by LEEH phase imaging could in future be used to extract chemical information about the imaged objects by mapping changes in scattering strength encoded by variations in the mean inner potential. Additionally, the exploration of charge distributions could be of particular interest in the context of ES-IBD sample preparation, for example to answer questions regarding whether the charge present in the gas phase is retained upon landing on the surface.

## Outlook

In the last 5 years, ES-IBD+LEEH has emerged as a promising single-molecule imaging technique, capable of revealing the shape of individual proteins at sub-nanometre resolution. At its present performance, it can provide complementary information to the well-established ensemble averaging methods, for example regarding conformational variability. However, many fundamental questions remain to be addressed, both on the experimental and on the theoretical levels. Instrumentation will have to be further developed to increase the obtainable resolution, for example via measurements at cryogenic temperatures, which are conducive to increased emitter stability, enhanced coherence [99], and a reduced energy spread of the electron beam [100]. Further improvements might include better detectors and an optimisation of the emitter-preparation process. In addition, the development of advanced reconstruction algorithms, which can provide a deeper understanding of LEEH phase maps as well as extract the three-dimensional information contained in the hologram, thereby allowing for three-dimensional imaging on the single-molecule scale, is of the utmost importance. Finally, the potential of preparative mass spectrometry techniques in LEEH sample preparation needs to be further explored. An increased understanding and control over the gas-phase conformations is needed to ensure the retention of native protein conformations during the sample-preparation process. The use of ion mobility filtering during the sample-preparation process would allow for a higher selectivity in the deposited conformations and thus enable correlated measurements of gas-phase conformations both by a collisional cross-section analysis and by direct LEEH imaging.

## Summary

- LEEH in conjunction with sample preparation performed by ES-IBD can be employed as a tool for imaging biomolecules at the single-molecule level at sub-nanometre resolution.
- LEEH imaging can map the conformational space of molecules with a high degree of conformational variability and thus provide insights both into the molecules’ native structure and their gas-phase structure.
- Phase retrieval methods can elucidate further structural detail related to the molecules’ mean inner potential as well as the presence of localised electric fields.

## Abbreviations

cryo-EM: electron cryo-microscopy
ES-IBD: electrospray ion beam deposition
LEEH: low-energy electron holography
PDB: protein data bank
SLG: single-layer graphene
TEM: transmission electron microscopy
UHV: ultrahigh vacuum
